# Dicer1 Promotes Colon Cancer Cell Invasion and Migration Through Modulation of tRF-20-MEJB5Y13 Expression Under Hypoxia

**DOI:** 10.3389/fgene.2021.638244

**Published:** 2021-03-08

**Authors:** Na Luan, Yali Mu, Jiayi Mu, Yiquan Chen, Xun Ye, Qin Zhou, Miaorong Xu, Qun Deng, Yeting Hu, Zhe Tang, Jianwei Wang

**Affiliations:** ^1^Department of Colorectal Surgery, 4th Affiliated Hospital, Zhejiang University School of Medicine, Hangzhou, China; ^2^Key Laboratory of Cancer Prevention and Intervention, Ministry of Education, Department of Colorectal Surgery and Oncology, 2nd Affiliated Hospital, Zhejiang University School of Medicine, Hangzhou, China; ^3^Department of Surgery, 4th Affiliated Hospital, Zhejiang University School of Medicine, Hangzhou, China; ^4^Department of Surgery, 2nd Affiliated Hospital, Zhejiang University School of Medicine, Hangzhou, China

**Keywords:** colorectal cancer, Dicer, tRNA-derived fragments, epithelial-to-mesenchymal transition, hypoxia

## Abstract

Hypoxia plays a key role in colorectal cancer (CRC) metastasis, but its underlying mechanism remains largely unknown. Dicer1, an RNase, has been considered as a tumor regulator in many tumors. However, whether Dicer1 affects CRC progression under hypoxia remains uncertain. In this study, we found that Dicer1 expression was induced by hypoxia in CRC cells and it mediates hypoxia-induced CRC cell progression. Furthermore, we found that the expression of tRF-20-MEJB5Y13, a small non-coding RNA derived from tRNA, was increased under hypoxic conditions, and its upregulation by Dicer1 resulted in hypoxia-induced CRC cell invasion and migration. These results advance the current understanding of the role of Dicer1 in regulating hypoxia signals and provide a new pathway for the development of therapeutic interventions for inhibiting cancer progression.

## Introduction

Colorectal cancer (CRC) is one of the most common causes of cancer-related deaths worldwide, which poses serious threats to public health, and its morbidity and mortality rates are increasing annually. Approximately 1.2 million new cases are reported annually, accounting for 10–15% of all malignant tumors, and it ranks third with respect to morbidity and mortality among malignant tumors (Miller et al., 2019). However, many clinical CRCs show micrometastasis of the tumor before surgery, which in itself is a complex and continuous process regulated by multiple factors and steps (Crotti et al., 2017). Therefore, studying the underlying mechanism of colon cancer invasion and migration is essential for establishing targeted intervention strategies to improve survival.

Tumor cells are characterized by uncontrolled cell proliferation, resistance to apoptosis, and metabolic shift to anaerobic glycolysis (Warburg effect). As the tumor cells continue to proliferate and the tumor volume increases, high demand for oxygen is created and this oxygen demand of the cells cannot be satisfied by the blood supply, which limits the use of oxygen by the cells, thereby resulting in hypoxic conditions for tumor cells (Catalano et al., 2013; Sormendi and Wielockx, 2018). Hypoxia, oxidative stress, and acidosis in the tumor microenvironment trigger extracellular matrix remodeling and stimulate adjacent stromal cells (such as fibroblasts) and immune cells (such as lymphocytes and macrophages) to induce angiogenesis, thereby promoting tumor invasion and migration ultimately (Roma-Rodrigues et al., 2019).

Recent studies have shown that Dicer1 required for transfer RNA-derived fragments (tRF) biogenesis is associated with the development of cancer (Karube et al., 2005; Chiosea et al., 2006, 2007; Flavin et al., 2008; Merritt et al., 2008; Grelier et al., 2009). Currently, several studies have shown that the expression level and mode of action of Dicer1 may vary depending on the type of tumor, but the regulatory mechanism of these genes remains unclear. Additionally, recent studies on CRC have demonstrated that high expression of Dicer1 mRNA and protein is associated with poor survival, and this effect is independent of any clinical parameters of patients with CRC, including sex and age (Chiosea et al., 2006). Interestingly, Dicer1 downregulation was statistically correlated with tumor progression, tumor grade, and lymph node metastasis (Faggad et al., 2012). However, the relationship between Dicer1 and tumor invasion and migration and their underlying mechanisms are unclear.

tRF belongs to the short non-coding RNA family present in most organisms, and can be processed by Dicer (Cole et al., 2009) and RNase (Lee et al., 2009) under stress conditions such as hypoxia. Dicer is a ribonuclease, which is generally considered to be the cause of mature miRNA biogenesis. A study suggests that Dicer can cleave tRNA from the 3′-end of tRNA to produce tRF (tRF-3) (Haussecker et al., 2010). Contrastingly, a recent study demonstrated that certain tRF-3 series can be produced independently of the Dicer enzyme (Kuscu et al., 2018). Dicer can also cleave the D arm of mature tRNA in cancer cells to produce tRF-5 (Cole et al., 2009). As hypoxia is the main stress condition encountered by the cells during cancer progression, tRF expression induced under hypoxia may affect metastasis (Goodarzi et al., 2015). Huang et al. (2017) identified a human-specific tRF/miR fragment, tRF/miR-1280, which could inhibit Notch/Gata and miR-200b signal transduction through direct interaction with JAG2 3′-untranslated region, thereby suppressing colon cancer cell proliferation and metastasis. Zhang et al. (2019) found that tRF-03357 might promote the proliferation, invasion and migration of SK-OV-3 cells.

Our aim was to explore whether hypoxia microenvironment affects Dicer1 expression in CRC cells and to investigate the relationship between changes in Dicer1 expression and epithelial-to-mesenchymal transition (EMT) in CRC. Finally, we identified a novel mechanism that links hypoxia with upregulated Dicer1 expression and specific tRF expression associated with EMT in CRC.

## Materials and Methods

### Cell Culture

The human CRC cell lines SW480 and RKO were obtained from the American Type Culture Collection. Cells were cultured in Dulbecco's modified Eagle medium (Gibco, USA) supplemented with 10% fetal bovine serum (BI, USA) and 100 IU/mL penicillin. All cells were incubated in a CO_2_ constant temperature incubator (37°C, 21% O_2_, and 5% CO_2_) (Thermo Fisher Scientific, Rockford, IL, USA) for normal oxygen culture or in a hypoxic incubator (37°C, 1% O_2_, and 5% CO_2_) for hypoxic culture for 24 h.

### Plasmid Construction and Cell Transfection

To construct the Dicer1 expression plasmid, the synthetic Dicer1 gene encoding the coding sequences was inserted into the pcDNA3.1 vector, and puromycin resistance gene and green fluorescent protein tag were added simultaneously. Lipofectamine 2000 was used to transfect RKO and SW480 cells with Dicer1 overexpression plasmid. tRF-20-MEJB5Y13 mimics and negative control mimics were purchased from (GenePharma Shanghai, China). For each dish of cells, 2 μL of Lipo 2000 was added to 48 μL of Opti-MEM and mixed. The plasmid preparation solution was added to the Lipo 2000 preparation solution in an equal volume and incubated for 20 min. After 2 h of transfection, the cells were harvested for further experiments.

### RNA Sequencing

Total RNA was extracted with Trizol (Invitrogen, Carlsbad, CA, USA). RNA integrity was analyzed with Agilent 2100 Bioanalyzer (Agilent Technologies, Santa Clara, CA, USA). Qubit RNA detection kit deteced RNA concentration in the Qubit Fluorometer (Invitrogen, Carlsbad, USA). The total RNA samples used in subsequent experiments all meet the following requirements: RNA integrity number (RIN) 7.0, 28S:18S ratio 1.5. The sequencing library was generated and sequenced by Boao Biotechnology (Beijing, China). The total dose of each sample is 5μg RNA. Briefly, ribosomal RNA (rRNA) was removed using the Ribose Zero Magnetic Kit (epicenter technologies, Madison, WI, USA). To remove linear RNA, total RNA was digested with RNase R (Epicenter Technologies, Madison, WI, USA). The NEBNext Ultra RNA Library Preparation Kit from Illumina (Nebraska, USA) was used to construct a sequencing library according to the manufacturer's protocol. In NEBNext first-strand synthesis reaction buffer (5x), RNA is fragmented into fragments of approximately 300 base pairs (bp) in length. The RNA fragment was synthesized by reverse transcriptase and random hexamer primers to synthesize the first strand cDNA, and the second strand cDNA was synthesized with 10x dUTP Mix in the second strand synthesis reaction buffer. The end of the cDNA fragment undergoes an end repair process, which involves adding a single a base and then connecting the adaptor. After connecting the Illumina sequencing adapter, the second strand of cDNA was digested with user enzyme (NEB, USA) to construct a strand-specific library. In order to amplify the library DNA, the library was purified and PCR enriched. Then, these libraries were certified by Agilent 2100 and quantified using the KAPA Library Quantification kit (KAPA Biosystems, South Africa). Finally, the library was sequenced with paired ends on an Illumina HiSeq sequencer (Illumina, San Diego, CA, USA). The read length of the paired ends was 150 bp.

### Reverse Transcription Quantitative Polymerase Chain Reaction

The cells (transfected cell lines including RKO and SW480) were collected in a 1.5 mL RNase-free eppendorf tube, and 500 μL TRIzol reagent was added to ensure that the cells were fully lysed. After RNA extraction and concentration determination, cDNA was reverse transcribed using a cDNA Synthesis Kit (Thermo Fisher Scientific). RT-qPCR amplifications were performed with the SYBR Green qPCR Master Mix (ABI) by using the ABI 7300 real-time PCR detection system. Detailed information including forward primers and reverse primers of specific tRFs and Dicer1 were designed and synthesized (Table 1).

**Table 1:** Primer sequences for qRT-PCR.

**Gene**	**Primer Sequence (5^**′**^-3^**′**^)**
Primer Sequence (5′-3′)	F: GTCGTGCCGTATTGGTAGTT
	R: CTGCTGTCGCTCATATGGTT
tRF-20-MEJB5Y13	F: AGGTATTCGCACT
	R: CGGATCAGAAGATT
U6	F: CTCGCTTCGGCAGCACA
	R: AACGCTTCACGAATTTGCGT

### Western Blot Assay

The transfected cells were collected in an eppendorf tube, and RIPA buffer (Sigma-Aldrich, Shanghai, China) was added to the lysate containing proteinase inhibitors depending on the density of cells. After quantitative analysis, the proteins were separated using sodium dodecyl sulfate-polyacrylamide gel vertical electrophoresis. The proteins were then transferred onto polyvinylidene fluoride (PVDF) membranes (Millipore, USA). The PVDF membranes were at the anode and the gel was at the cathode electrode, and the apparatus was then immersed in the electrophoresis tank. Subsequently, the PVDF membranes were immersed in a blocking solution containing 5% skimmed milk powder and sealed at room temperature for 2 h. Thereafter, the blocked PVDF membranes were removed, immersed in 1 × phosphate-buffered saline solution with Tween 20, washed 3 times on a shaker, diluted with primary antibody solution, and incubated at 4°C overnight. The following primary antibodies were used: anti-N-cadherin [1:1000, Cell Signaling Technology (CST), USA], anti-vimentin (1:1000, CST, USA), anti-ZEB1 (1:1000, CST, USA), anti-MMP7 (1:1000, CST, USA), anti-Slug (1:1000, CST, USA), and anti-SSnail (1:1000, CST, USA). Subsequently, the membranes were probed with goat anti-rabbit IgG antibody (1:3000, Sigma-Aldrich, Japan). The proteins were then visualized with enhanced chemiluminescence western blot detection reagents (Bio-Rad, Cal, USA) and exposed to the chemical photosensitive mode.

### Transwell Assay

Cell migration and invasion assays were conducted using 24-well Transwell® chambers (Costar, USA). Serum-free medium was added to each well in order to adjust the densities of RKO and SW480 cells to 1.6 × 10^5^ cells/mL before seeding the cells in the upper chamber, whereas 500 μL of fetal bovine serum-supplemented medium was added to the lower layer, and then the chamber was incubated in a 37°C, 5% CO_2_, saturated humidity incubator for 2 h or overnight. For invasion experiments, it is necessary to pre-lay 20 μL of Matrigel mixture (the ratio of Matrigel and Dulbecco's modified Eagle medium was 1: 1) on the upper layer of the chamber in advance and incubate it at 37°C for 30 min until the glue solidifies. Matrigel needs to be melted into a liquid state beforehand, and it is not necessary to add matrigel for migration experiments. After culturing the cells at 37°C for 24 h, the cells were fixed with methanol for 20 min, washed with phosphate buffered saline 3 times, and stained with crystal violet. The cell number was visually counted in 3–5 random fields by using an inverted fluorescence microscope (Leica DMI3000B).

### 3-(4,5-Dimethylthiazol-2-yl)-2,5-Diphenyltetrazolium Bromide (MTT) Assay

MTT assay was performed to measure the proliferation of CRC cells. The transfected cells were cultured in 96-well-plates, and 96-well-flat bottom plates were incubated at 3°C for 24, 48, 72, and 96 h. Thereafter, 50 μL MTT solution (Sigma-Aldrich, St. Louis, MO) was added to the cells after the incubation, and the plates were then incubated for 4 h in a cell culture incubator under dark conditions to form formazan. Subsequently, 150 μL of dimethyl sulfoxide was added to each well. The density of cells in each well was measured by taking the absorbance at a wavelength of 570 nm.

### Statistical Analysis

All data are expressed as mean ± standard deviation. Data analysis was performed using GraphPad Prism 7 and SPSS 22.0 (version 22.0; IBM SPSS, Armonk, NY, USA) statistical softwares. All experiments were repeated 3 times. Statistical comparisons for significance were performed using the Wilcoxon signed-rank test for paired samples. Differences between groups were analyzed statistically using the paired Student's *t*-test. *P* < 0.05 were considered statistically significant.

## Results

### Hypoxia Inhibits Tumor Growth but Promotes Cell Invasion and Migration

**Figure 1:**
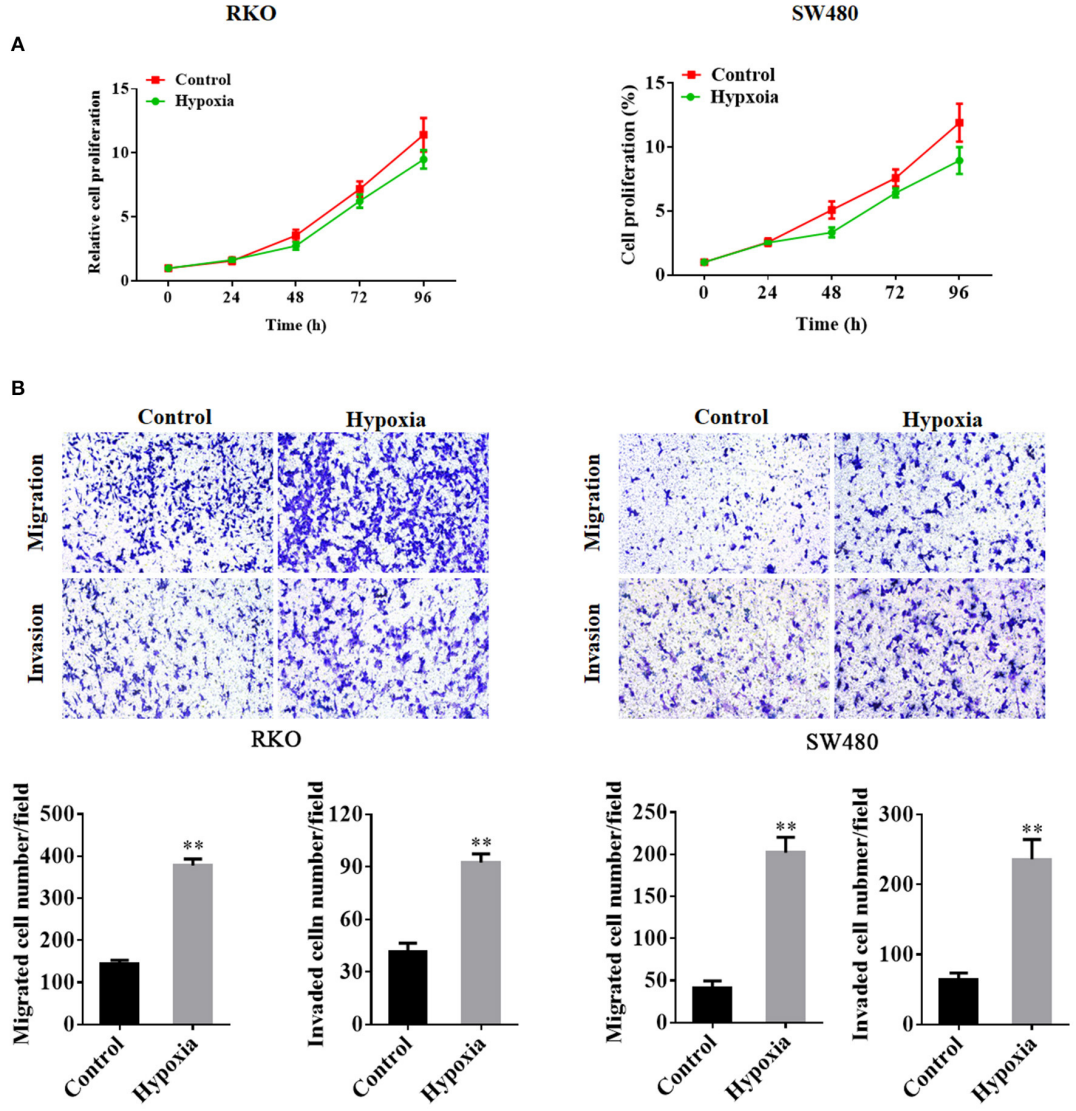
Colorectal cancer cell proliferation, migration, and invasion under nomoxic and hypoxic conditions. The MTT proliferation assay was performed to determine cell proliferation **(A)**. Transwell assays were performed to detect the migration and invasion of RKO cells and SW480 cells under normoxic or hypoxic conditions **(B)**. **P < 0.01. The assays were repeated at least 3 times. MTT, 3-(4,5-dimethylthiazol-2-yl)-2,5-diphenyltetrazolium bromide.

Cell proliferation in the hypoxia group was decreased as compared to that in the control group (Figure 1A). However, the transwell assays showed that the number of migrated and invaded cells was significantly increased in the hypoxic groups in RKO and SW480 cells (Figure 1B).

### Dicer1 Was Upregulated in CRC Cells Under Hypoxic Conditions

**Figure 2:**
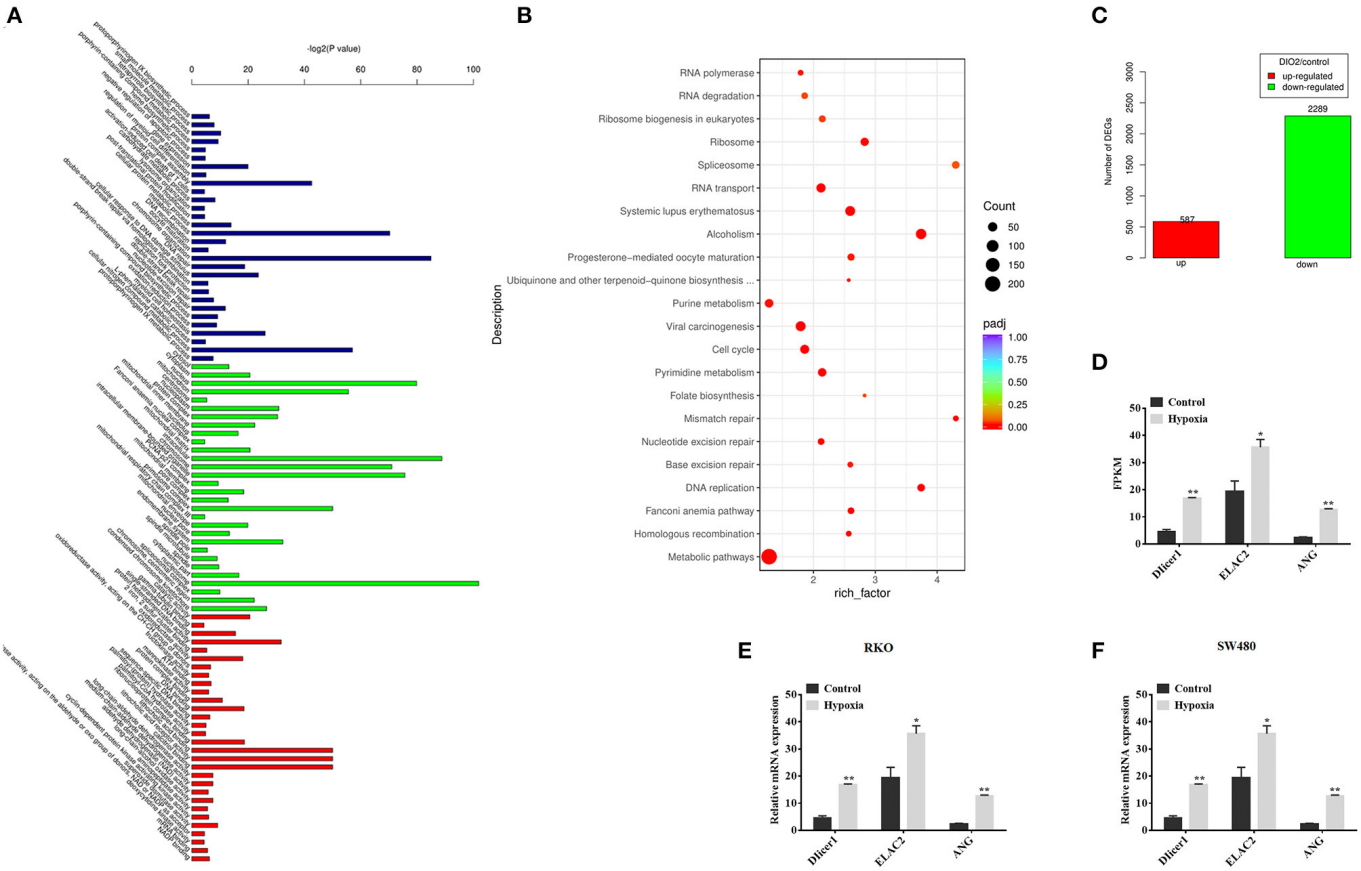
Dicer1 upregulation in colorectal cancer cells under hypoxic conditions. GO terms listed can be clustered into three groups: (I) biological process, (II) molecular function, and (III) cellular component, describing detailed biological processes in their respective levels **(A)**. Apart from the GO terms, Bulb map of KEGG analysis was found to be effective for identifying different genes expressed under hypoxic and normoxic conditions. Rich factor represented the enrichment degree of differentially expressed genes. Y axis showed the name of enriched pathways. The area of each node represented the number of the differentially enriched host genes. The *p*-value was represented by a color scale. The statistical significance increased from purple (relatively lower significance) to orange (relatively higher significance) **(B)**. Sequencing analysis results showed significant differences of gene expression **(C)**. High-throughput sequencing was performed to detect the expression of RNases, which plays an important role in the biological process of tRFs, between the two groups **(D)**. The RT-qPCR assay results verify the expression of Dicer1 in RKO and SW480 cells **(E,F)**. *P < 0.05, **P < 0.01. The assays were repeated more than 3 times. NC, negative control; GO, Gene Ontology; KEGG, Kyoto Encyclopedia of Genes and Genomes; tRF, transfer RNA-derived fragments; RT-qPCR, reverse transcription quantitative polymerase chain reaction.

The relationship between Dicer and cancer prognosis has been observed in many human cancers. However, whether Dicer plays a functional role in the mechanism of CRC progression under hypoxic conditions and whether they are associated with tRFs remains to be elucidated. To test our hypothesis, we performed high-throughput sequencing to identify changes in the expression of Dicer1 and tRFs. Next, we successfully sequenced small RNAs from CRC cells under hypoxic conditions. GO terms listed can be clustered into three groups: (I) biological process, (II) molecular function, and (III) cellular component, describing detailed biological processes in their respective levels (Figure 2A). Enrichment analysis of the KEGG signaling pathway suggested that RNA degradation, RNA transport and Splisome were significantly enriched in the signaling pathways (Figure 2B). According to the sequencing results, the number of genes with significant differences in expression were identified. Among them, 888 genes were significantly upregulated and 3,168 genes were significantly downregulated under hypoxic conditions (Figure 2C). The expression of 3 RNases involved in tRNA cleavage were found to be increased. Notably, the expression of the RNase Dicer, which plays an important role in the biological process of tRFs, varied significantly between the two groups (Figure 2D). The expression of Dicer1, which increased significantly under hypoxic conditions, was further evaluated using RT-qPCR analysis. RT-qPCR results showed that Dicer1 mRNA expression levels were significantly upregulated in RKO and SW480 cells under hypoxic conditions (Figures 2E,F). The data showed that RT-qPCR results were consistent with the expression patterns identified through high-throughput sequencing.

### Dicer1 Overexpression Promotes CRC Cell Invasion and Migration

**Figure 3:**
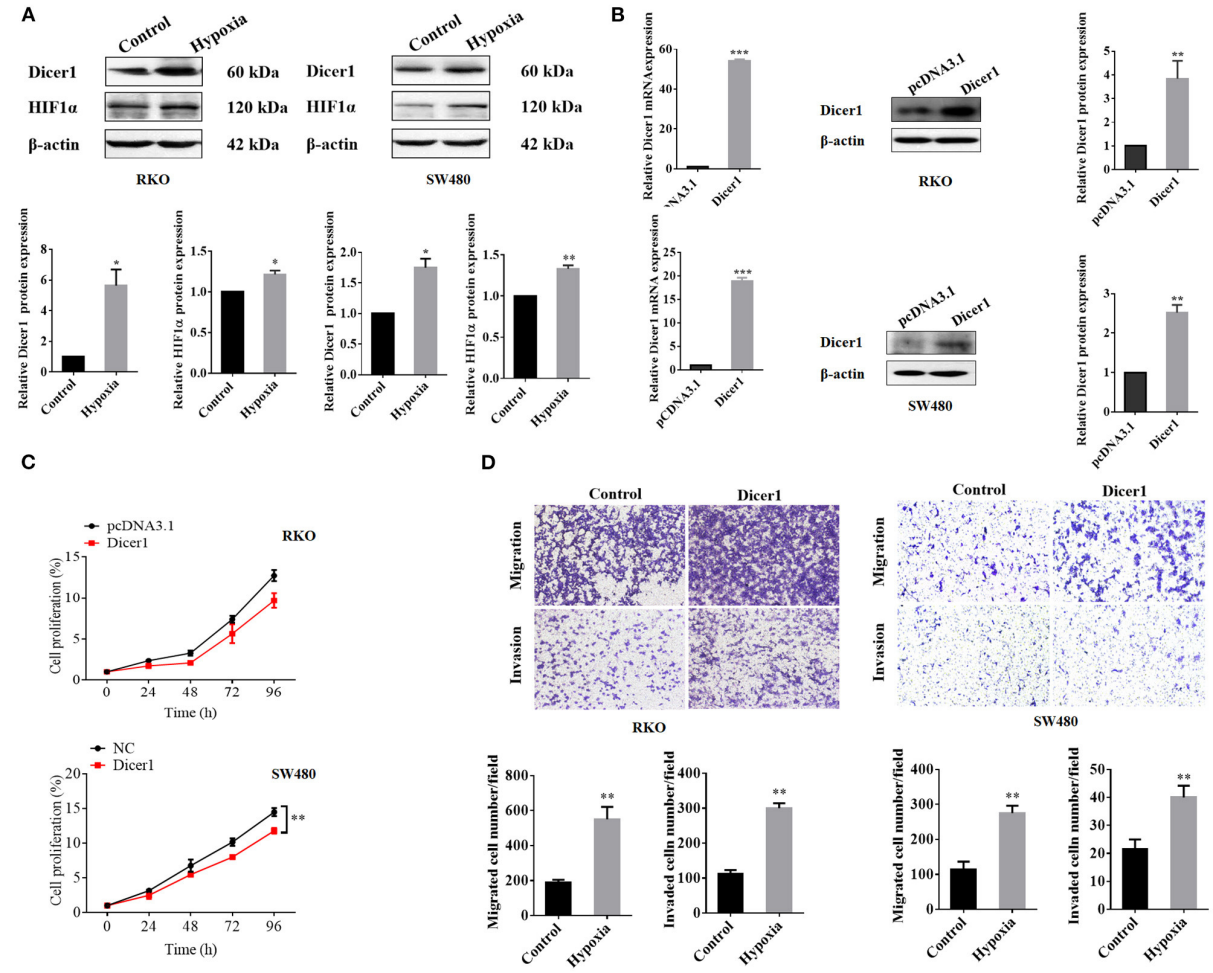
Upregulation of Dicer1 expression affects the proliferation, invasion, and migration of colorectal cancer cells. Dicer1 and HIF1α expression under hypoxic conditions was further evaluated using western blot assays **(A)**. We transfected the Dicer1 overexpression plasmid into RKO and SW480 cells and detected its transfection efficiency using RT-qPCR and western blot assays **(B)**. MTT test results showed the proliferation of RKO and SW480 cells **(C)**. Transwell assays showed that Dicer1 overexpression could promote RKO and SW480 cell invasion and migration **(D)**. *P < 0.05, **P < 0.01, and ***P < 0.001. The assays were repeated more than 3 times. NC, negative control; RT-qPCR, reverse transcription quantitative polymerase chain reaction; MTT, 3-(4,5-dimethylthiazol-2-yl)-2,5-diphenyltetrazolium bromide.

The expression of Dicer1, which increased significantly under hypoxic conditions, was further evaluated through western blot assays. The results showed that Dicer1 protein expression was significantly upregulated in RKO and SW480 cells under hypoxic conditions (Figure 3A). Furthermore, RKO and SW480 cells were used for subsequent studies. After transfecting Dicer1 overexpression plasmid in RKO and SW480 cells with Lipofectamine 2000, the transfection efficiency was estimated using RT-qPCR and western blot assays. The mRNA and protein expression of Dicer1 in the transfected group were evidently higher than those in the negative control group (Figure 3B). Next, the MTT assay was performed to study the effects of Dicer1 on CRC cell proliferation. The MTT assay results suggested that Dicer1 upregulation significantly decreased the proliferation of RKO and SW480 cells (Figure 3C). Furthermore, we performed transwell assays to investigate whether Dicer1 overexpression could influence CRC cell invasion and migration. The assay results showed that Dicer1 upregulation evidently promoted RKO and SW480 cell invasion and migration in the transfected groups as compared to the negative control groups (Figure 3D). Taken together, these findings indicate that Dicer1 plays a critical role during CRC development.

### Dicer1 Overexpression in CRC Cells Increases the Expression of Cell Invasion and Migration Markers

**Figure 4:**
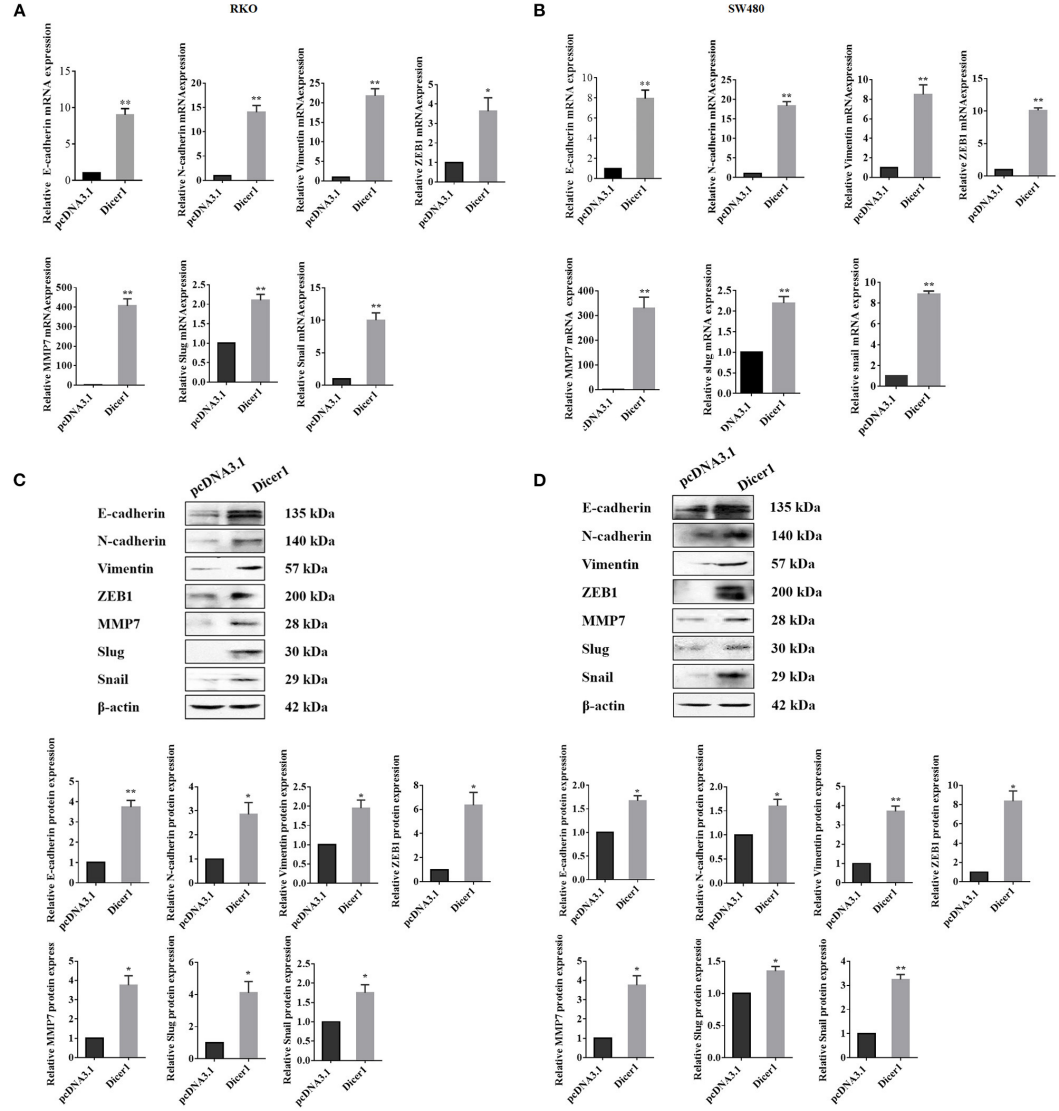
Increased Dicer1 expression in colorectal cancer cell lines promotes the expression of various carcinogenic markers, including EMT-related markers. Comparison of the mRNA expression of indicated markers in RKO and SW480 cells between the control and Dicer-overexpressing groups based on RT-qPCR analysis **(A,B)**. Western blot assays were performed for analyzing the expression of E-cadherin, N-Cadherin, Vimentin, ZEB1, MMP7, Slug, and Snail **(C,D)**. *P < 0.05, **P < 0.01. The assays were repeated more than 3 times. RKO and SW480, human colorectal cancer cells; NC, Negative control; EMT, epithelial-to-mesenchymal transition; RT-qPCR, reverse transcription quantitative polymerase chain reaction.

We found that Dicer1 overexpression promotes CRC cell invasion and migration. Thereafter, we investigated whether upregulated Dicer1 expression could affect the mRNA and protein expression of oncogenic and EMT-related markers in RKO and SW480 cell lines. The results showed that both the mRNA and protein expression of MMP7 and EMT-related markers, including E-cadherin, N-cadherin, vimentin, ZEB1, Slug, and Snail were upregulated after Dicer1 transfection (Figures 4A–D). Therefore, the overexpression of these markers indicates an increased potential for cell invasion, migration, and metastasis following Dicer1 overexpression. Taken together, our results suggest that increasing Dicer1 expression markedly increased the expression of a broad range of tumor progression markers.

### Dicer1 Facilitated CRC Cell Migration and Invasion Through tRF-20-MEJB5Y13

**Figure 5:**
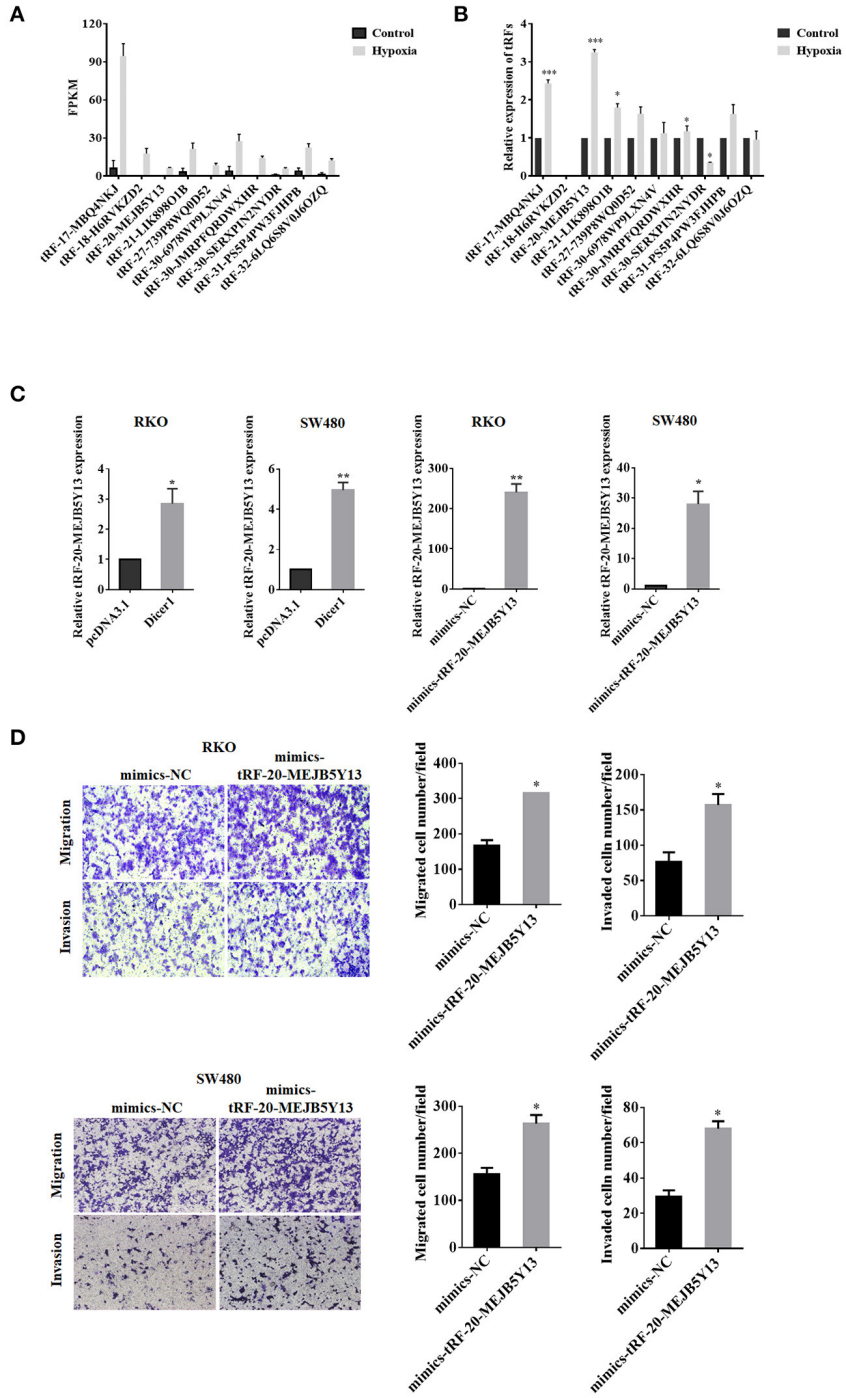
Dicer1 promotes the invasion and migration of CRC through tRF-20-MEJB5Y13. High-throughput sequencing and RT-qPCR analyses showed significant differences in the expression of 14 tRFs **(A,B)**. RT-qPCR assay was performed to explore whether tRF-20-MEJB5Y13 expression was related to Dicer1 overexpression and to detect the transfection of tRF-20-MEJB5Y13 mimics **(C)**. Transwell assays were performed to evaluate CRC cell migration and invasion with tRF-20-MEJB5Y13 overexpression **(D)**. *P < 0.05, **P < 0.01. The assays were repeated more than 3 times. CRC, colorectal cancer; RT-qPCR, reverse transcription quantitative polymerase chain reaction; tRF, transfer RNA-derived fragments.

When the expression of small RNAs in CRC cells under hypoxic conditions were compared with the control group, the expression of 14 tRFs was found to significantly vary between the two groups. High-throughput sequencing data revealed that tRF-20-MEJB5Y13 expression was significantly upregulated in CRC cells under hypoxic conditions (Figure 5A). The expression of tRF-20-MEJB5Y13, which increased significantly under hypoxic conditions, was further evaluated using RT-qPCR analysis. Additionally, PCR analysis suggested that tRF-20-MEJB5Y13 expression was consistent with the high-throughput sequencing data obtained for CRC cell lines under hypoxic conditions (Figure 5B). Several assays were performed to further confirm our hypothesis that Dicer1 promotes CRC cell invasion and migration through its cleavage product, tRFs, under hypoxic conditions. tRF-20-MEJB5Y13 expression was significantly upregulated with Dicer1 overexpression, thereby suggesting that the expression of Dicer1 and tRF-20-MEJB5Y13 was positively correlated, and Dicer1 may act through tRF-20-MEJB5Y13 (Figure 5C). Next, we further explored the effect of tRF-20-MEJB5Y13 on the progression of CRC cells. RT-qPCR analysis results revealed that tRF-20-MEJB5Y13 expression was significantly upregulated after transfecting the cells with tRF-20-MEJB5Y13 mimics (Figure 5C). Furthermore, transwell assay results indicated that tRF-20-MEJB5Y13 overexpression in RKO and SW480 cells enhanced cell migration and invasion, respectively (Figure 5D), thereby suggesting that tRF-20-MEJB5Y13 might play a critical role in promoting CRC cell migration and invasion.

**Figure 6:**
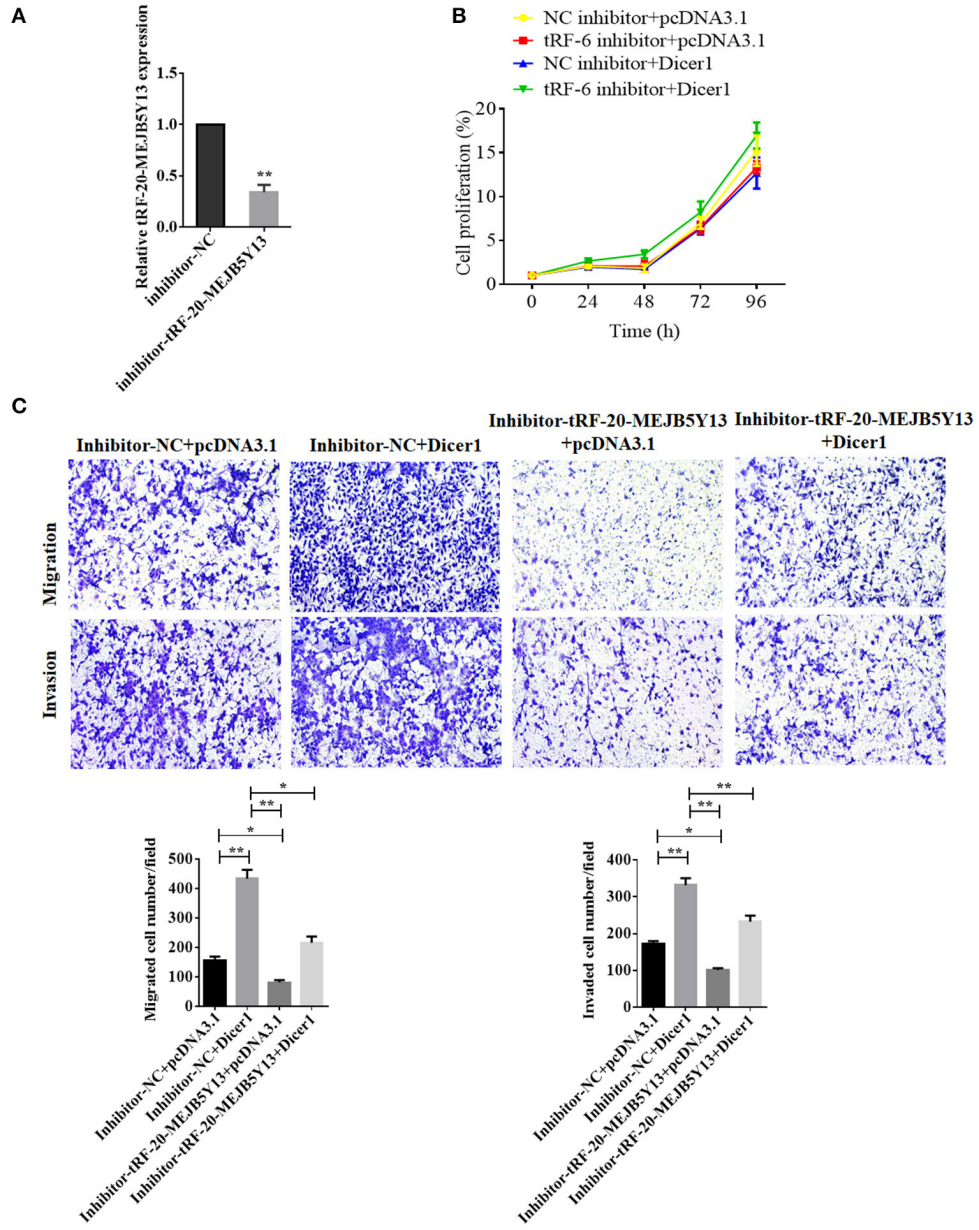
RT-qPCR assay was performed to detect the transfection of tRF-20-MEJB5Y13 inhibitor **(A)**. MTT assays were performed to explore CRC cell proliferation after the co-transfection of both Dicer1 mimics and tRF-20-MEJB5Y13 inhibitor **(B)**. Transwell assays were performed to evaluate the common effects of Dicer1 overexpression and tRF-20-MEJB5Y13 knockdown on CRC cell invasion and migration **(C)**. *P < 0.05, **P < 0.01. The assays were repeated more than 3 times. CRC, colorectal cancer; RT-qPCR, reverse transcription quantitative polymerase chain reaction; MTT, 3-(4,5-dimethylthiazol-2-yl)-2,5-diphenyltetrazolium bromide.

To confirm the influence of Dicer1 on the biological function of CRC and that it acts through tRF-20-MEJB5Y13, we stably overexpressed Dicer1 and inhibited tRF-20-MEJB5Y13 expression (Figure 6A). MTT assays were used to detect the effects upon combination of Dicer1 and tRF-20-MEJB5Y13 on proliferation of CRC cells. The results indicated that tRF-20-MEJB5Y13 could prompt cells proliferation, which partially covered up the inhibiting effects of Dicer1 (Figure 6B). Transwell assays were performed to detect the effect of Dicer1 combined with tRF-20-MEJB5Y13 on CRC cell invasion and migration. Therefore, the results show that Dicer1 overexpression can significantly promote CRC cell invasion and migration, and this effect is partially eliminated by suppressing tRF-20-MEJB5Y13 expression (Figure 6C).

## Discussion

Dicer1 is a ribonuclease, a key enzyme necessary for the biogenesis of non-coding RNA such as microRNA, tRFs, and small interfering RNAs, and is essential for mammalian development and cell differentiation (Grelier et al., 2009). In the present study, we confirmed that CRC cell invasion and migration were increased under hypoxic conditions. Our results showed that Dicer1 expression under hypoxic conditions in colon cancer cells was upregulated. Moreover, the results suggested that Dicer1 enhances the invasion and migration ability of CRC cells. More importantly, we found that Dicer1 mediated EMT through tRF-20-MEJB5Y13, a key mechanism that promotes CRC cell invasion and migration.

Rapid proliferation of cancer cells as well as structural and functional defects in tumor blood vessels results in hypoxic conditions within solid tumors (Gilkes et al., 2014). Hypoxia, a characteristic of the tumor microenvironment and a common feature of solid tumors, has been associated with an increased risk of tumor metastasis and poor prognosis (Hiraga, 2018). Our study verified that hypoxic conditions inhibit the growth of CRC cells. We speculate that hypoxia may cause tumor cells to initiate apoptosis or cell necrosis. It is known that the genes *bnip3*, Bcl-2/adenovirus EIB 19 kDa-interacting protein 3, and *bnip3L* (bnip3-like), whose products are members of the Bcl-2 homology 3-only protein family of cell death factors, are highly expressed in hypoxia. Furthermore, a large amount of data shows that hypoxia promotes the invasion potential of tumor cells. Hypoxia-inducible factor activation is associated with the loss of E-Cadherin, a component of the meeting point of believers that acts as an inhibitor of invasion and migration (Sullivan and Graham, 2007). Interestingly, TWIST1 regulates EMT and is induced under hypoxia (Yang et al., 2006). In addition, cells that survive acidosis not only proliferate, but also become more aggressive and invasive (Walenta and Mueller-Klieser, 2004). This effect is partly through the activation of hypoxia-inducible factor-UPD regulatory proteins involved in matrix remodeling, such as Lysyl oxidase (Petrella et al., 2005), and metalloproteinases that disrupt extracellular matrix interactions (Pouyssegur et al., 2006).

To explore the specific mechanism of increased invasion and migration ability of CRC cells under hypoxic conditions, we analyzed and compared the expression of different genes between the test and the control groups. KEGG analysis results revealed that the RNA degradation and spliceosome pathways were enriched in CRC cells. Hence, in this study, we examined the differences in Dicer1 expression between hypoxic and normoxic conditions in CRC cells to explore whether Dicer1 expressional changes are related to CRC progression. High-throughput sequencing and RT-qPCR analyses conducted on CRC cell lines cultured under normoxia and hypoxia demonstrated that Dicer1 expression is upregulated under hypoxic conditions. However, Lai et al. found that hypoxia-inducible factor 1α (HIF1α) induced the proteolysis of Dicer1 in the CRC cell line HCT116 (Lai et al., 2018). While presumably other pathways or regulatory mechanisms that lead to the increased expression of Dicer1 under hypoxic conditions, since Dicer1 is a specific nuclease that produces tRFs from tRNA cleavage under hypoxia (Shen et al., 2018). Although Dicer1 displays different expression patterns in different cancers, our data suggest that Dicer1 expression under hypoxic conditions in colon cancer cells was upregulated and it may affect CRC cells and tumor microenvironment.

Several studies have been carried out on different types of cancer to clarify the role of Dicer1 in carcinogenesis and its impact on prognosis. In the present study, proliferation assays showed that Dicer1 transfection inhibited the proliferation of two CRC cell lines. Our transwell assay results suggested that upregulated Dicer1 expression promotes CRC cell invasion and migration. Our results were consistent with the results reported for prostate adenocarcinoma, which suggested that Dicer1 expression was upregulated and affected tumor invasion characteristics (Chiosea et al., 2006). Consistently, a study investigating the expression of ribonuclease Dicer in CRC cell lines reported that Phase III Dicer mRNA expression was significantly higher than that in the Phase II, thereby suggesting that Dicer may play a key role in the development of a more invasive form of tumor (Papachristou et al., 2011). In ovarian cancer, Dicer expression correlates with lymph node metastasis status, and its high expression denotes lymph node metastasis (Flavin et al., 2008). However, on the contrary, in breast cancer cell lines, low Dicer expression was found in cells with mesenchymal phenotypes and metastatic bone derivatives, thereby indicating that downregulated Dicer expression may be related to tumor metastasis (Grelier et al., 2009). Moreover, Dicer has a tumor-suppressing effect on lung adenocarcinoma and ovarian cancer cells (Chiosea et al., 2007; Merritt et al., 2008). Although Dicer plays different roles in the progression of cancer in different solid tumors, our study found that upregulated Dicer1 expression is associated with CRC cell invasion and migration. Therefore, Dicer1 overexpression may be a powerful independent predictor of poor CRC prognosis.

Next, we explored the mechanism through which Dicer1 promotes CRC cell invasion and migration. The most common mechanism for enhancing the motility of cancer cells is EMT, which liberates tumor cells from adhesion, thereby allowing them to migrate and invade. In our study, we confirmed that Dicer1 overexpression induced the expression of EMT-related molecules, namely E-cadherin, N-cadherin, vimentin, ZEB1, MMP7, Slug, and Snail. This suggests that the elevated expression of Dicer1 in CRC cells contributes to multiple carcinogenic characteristics, including cell migration and invasion.

Notably, Dicer1 is the core component of the tRF regulatory network. Therefore, several studies have aimed to explore the significant role of ribonuclease in cancer pathology. In the present study, high-throughput sequencing and bioinformatics analysis results revealed significant differences in the expression of 14 tRFs under hypoxic conditions (named from database MINTbase). Retrospective verification results of RT-qPCR experiments showed that the expression of tRF (tRF-20-MEJB5Y13) in the differentially expressed tRFs was significantly upregulated with the most significant differences in their expression, which was consistent with that of the sequencing results. Recently, it was reported that the expression of a series of tRFs is altered in cancer tissues, and they are related to the development of cancer (Chen et al., 2019). Huang et al. found that tRF/miR-1280 expression was decreased in tumor tissues as tRF/miR-1280 overexpression reduces cell proliferation and colony formation (Huang et al., 2017). In our study, we explored the specific role and regulatory mechanism of tRF-20-MEJB5Y13 in the EMT in CRC. Functional assays verified that tRF-20-M0NK5Y93 overexpression can facilitate the acquisition of a variety of oncogenic characteristics, including cell invasion and migration, which supported the hypothesis that tRF-20-MEJB5Y13 functions as a tumor-suppressive molecule *in vitro*. These findings demonstrate that tRF-20-MEJB5Y13 exerts its effects on CRC progression by promoting the metastatic activity of CRC cells.

Finally, our results showed that tRF-20-MEJB5Y13 knockdown could inhibit the stimulatory effect of Dicer1 overexpression on CRC cell migration and invasion. Thus, our results suggest that Dicer1 plays a key role in CRC progression by regulating tRF-20-MEJB5Y13 expression under hypoxic conditions. Currently, we have reasons to believe that Dicer1 acts as a nuclease to produce tRF-20-MEJB5Y13 from pre-tRNAs and mature tRNAs, thereby promoting CRC cell invasion and migration. However, there are other reasons why high Dicer1 expression makes CRC cells more invasive. Dicer performs additional functions that may lead to malignant transformation. Recently, studies have shown that Dicer1 is essential for maintaining the methylation of CpG promoter islands in CRC cell lines (Ting et al., 2008). As we know, over-methylation and under-methylation of several genes in CRC are common phenomena, and Dicer can promote or at least maintain these carcinogenic events (Samowitz and Ogino, 2008). Therefore, this may partly explain the contradiction between the findings of colorectal cancer and other cancers.

## Conclusion

In this study, we found that Dicer1 expression can be induced by hypoxia in CRC cells, and it promotes hypoxia-induced CRC cell metastasis. Additionally, we found that the expression of tRF-20-MEJB5Y13, a small ncRNA, was increased under hypoxic conditions, and its upregulation by Dicer1 resulted in hypoxia-induced CRC cell progression. Therefore, further studies need to be performed to establish Dicer1 as a potential tumor metastasis molecule, thereby further developing it as a novel biological marker for tumor development diagnosis or as a new avenue for new drugs, which offers an innovative strategy for inhibiting tumor metastasis that has broad application prospects in cancer advances.

## Data Availability Statement

The original contributions presented in the study are included in the article/Supplementary Material, further inquiries can be directed to the corresponding author/s.

## Author Contributions

JW and ZT provided the idea and designed the framework of the study. NL, YM, and JW conceived and designed the experiments. NL, YM, JM, XY, QZ, and MX performed the experiments. NL, QD, YH, and JW analyzed the bioinformatics data. NL wrote the draft. YH, ZT, and JW revised the manuscript. All authors have read and approved the final manuscript.

## Conflict of Interest

The authors declare that the research was conducted in the absence of any commercial or financial relationships that could be construed as a potential conflict of interest.

## Abbreviations

CRC: Colorectal cancer
tRFs: tRNA-derived fragments
EMT: Epithelial-to-mesenchymal
RT-qPCR: Reverse transcription quantitative polymerase chain reaction
PVDF: Polyvinylidene fluoride
CST: Cell Signaling Technology
MTT: 3-(4,5-Dimethylthiazol-2-yl)-2,5-diphenyltetrazolium bromide.
